# Pesticide and Pathogen Exposure Causes Idiosyncratic Gene Expression Responses Across Four Diverse North American Bumble Bee Species

**DOI:** 10.1111/mec.70042

**Published:** 2025-08-08

**Authors:** Rubén Martín‐Blázquez, Sydney A. Cameron, Austin C. Calhoun, James P. Strange, Ben M. Sadd

**Affiliations:** ^1^ Department of Entomology University of Illinois Urbana‐Champaign Urbana Illinois USA; ^2^ School of Biological Sciences Illinois State University Normal Illinois USA; ^3^ Department of Entomology The Ohio State University Columbus Ohio USA

**Keywords:** *Bombus*, gene expression, pathogens, pesticides, RNA‐seq, species decline

## Abstract

Bumble bee (*Bombus* Latreille) populations of certain species have declined precipitously in North America over several decades. Hypotheses for declines include exposure to the pathogen *Nosema bombi* and neonicotinoid pesticides. Importantly, populations of some bumble bee species remain stable despite their presumed exposure to these same stressors. We hypothesise that declining and stable species exhibit distinct responses to *N. bombi* and neonicotinoids, detectable as differential gene expression profiles. To test this, we exposed larvae of *Bombus occidentalis* (declining) and *B. impatiens* (stable) to *N. bombi* and to the neonicotinoid imidacloprid, plus a combination of both. RNA‐seq analysis revealed almost no overlap between these species in gene expression responses to the individual stressors. There was more overlap of differentially expressed genes for the combined‐stressor condition, but hundreds of genes still showed species‐specific expression differences. To test whether the differential molecular responses could be associated with declining and stable species, we performed quantitative PCR on 20 selected genes, adding two additional species *B. terricola* (declining) and *B. griseocollis* (stable). These responses did not separate out by species decline status; each of the four species exhibit species‐specific responses. Overall, these results highlight that generalising mechanisms and causes of decline across different species may be misleading, as diverse species respond molecularly in a species‐specific manner to particular environmental stressors.

## 1 Introduction

Wild bumble bees (*Bombus* Latreille) play a critical role within a community of pollinators, contributing valuable ecosystem services through pollination of wild plants and crops in many regions of the world (Garibaldi et al. 2013; McGrady et al. 2021; Ollerton 2017). Widespread reports of native bumble bee species declines in range and relative abundance over the last few decades are therefore of great concern (Bommarco et al. 2012; Cameron et al. 2011; Cameron and Sadd 2020; Colla and Packer 2008; Giles and Ascher 2006; Jacobson et al. 2018; Morales et al. 2013; Rasmont et al. 2015). The declines, however, are not ubiquitous across all bumble bee species (Cameron and Sadd 2020), as some maintain relatively stable or expanding distributions (Ghisbain et al. 2021). Such species‐specific patterns have been especially well documented in North America where several species within the subgenus *Bombus sensu stricto*, including the federally endangered *Bombus affinis* , have exhibited striking declines (Cameron et al. 2011; Janousek et al. 2023), while species such as *B. impatiens* (subgenus *Pyrobombus*) are not only healthy (Cameron and Sadd 2020) but appear to be expanding their range (Looney et al. 2019). Yet after two decades of study, we still lack precise evidence of causal relationships in bumble bee health and what factors may be driving species‐specific differences.

Multiple factors have been reported as potential causal factors behind bumble bee species declines. Changes in land use and habitat fragmentation (Janousek et al. 2023; Straub et al. 2023), nutritional stress (Biesmeijer et al. 2006; Leza et al. 2018; Rundlöf et al. 2014; Woodard et al. 2019), climate change and extreme climate events (Jackson et al. 2022; Janousek et al. 2023; Soroye et al. 2020; Tobin et al. 2024), parasite and pathogen infection (Cameron et al. 2016; Colla et al. 2006; Cordes et al. 2012; Murray et al. 2013) and exposure to pesticides (Fauser et al. 2017; Janousek et al. 2023; McArt et al. 2017; O'Reilly and Stanley 2023) have all been proposed as potential factors behind North American species declines. Field and laboratory studies have documented how these factors may affect individual, population and community health. Moreover, the multiple‐stressor hypothesis posits that combinations of stressors exacerbate individual effects to the detriment of bumble bee health (Botías et al. 2021; Calhoun et al. 2021; Straub et al. 2023). The fact that there are documented stable and declining species that overlap in their ranges suggests differences in species susceptibility or differential localized exposure to these diverse stressors. Documented differences in species responses to certain stressors have been reported (Baron et al. 2017; Feuerborn et al. 2023; Martinet et al. 2015; Moerman et al. 2016; Oyen et al. 2016), but many such studies, both laboratory and field, extrapolate outcomes from a single stable, usually commercially reared, species (discussed in Cameron and Sadd 2020). As such, we lack information on whether and how wild bumble bee species with contrasting decline statuses vary in their responses to agents of decline in a way that could explain heterogeneity in long‐term population health throughout the genus.

In North America, the microsporidian pathogen *Nosema bombi* has been advanced as a likely factor linked to species‐specific bumble bee decline, as declining populations of most species in the subgenus *Bombus s. s*. and some species of *Thoracobombus* have been shown repeatedly to have significantly higher prevalences of *N. bombi* than stable species (Cameron et al. 2011; Cameron et al. 2016; Gillespie 2010). Infection is primarily established following larval exposure to environmentally resistant spores (Calhoun et al. 2021; Rutrecht et al. 2007). Individual health and colony fitness effects of infection have been demonstrated experimentally (Martinez et al. 2023; Otti and Schmid‐Hempel 2007, 2008; Rutrecht and Brown 2009) and closely related species may be differentially affected (Brown 2017), as reported from studies of differential infection impacts on *B. lucorum* and *B. terrestris* gynes (Rutrecht and Brown 2009). Moreover, *B. impatiens* males appear to have a high tolerance to experimentally established infections (Calhoun et al. 2021). No single study, however, has investigated how bumble bee species may differ in their molecular responses to *N. bombi* exposure.

In addition to the threat from pathogen infection, increased neonicotinoid pesticide use has received global attention in relation to insect decline generally (Goulson 2013) and to bumble bees specifically (Cameron and Sadd 2020; Godfray et al. 2015; Janousek et al. 2023). As systemic pesticides, neonicotinoids accumulate throughout tissues of plants (Main et al. 2017), and may contaminate natural plant populations from treated crop plants through soil percolation or pesticide drift (Berens et al. 2021; Mitchell et al. 2017; Rondeau and Raine 2024; Sánchez‐Bayo 2021; Thompson et al. 2020). Accumulation in nectar and pollen can expose nontarget beneficial insect pollinators to the harmful effects of these pesticides (Bonmatin et al. 2015; David et al. 2016). At the molecular level, insects may mitigate the effects of neonicotinoids in real time via detoxification by specific cytochrome P450 proteins (Chaimanee et al. 2016; Daborn et al. 2001), or on an evolutionary timescale through selection of resistant variants of this pesticide's target, the neonicotinoid acetylcholine receptors (as happens in some populations of *Drosophila* (Lu et al. 2022)). Despite reduced numbers of detoxification genes in their genomes relative to many insects (Sadd et al. 2015), bumble bees have cytochrome P450 and other genes able to (putatively) detoxify neonicotinoids (Haas et al. 2022) and these genes can be expressed with pesticide exposure (Manjon et al. 2018; Colgan et al. 2019; Witwicka, López‐Osorio, Arce, et al. 2025). Nevertheless, multiple sublethal effects of exposure to neonicotinoids have been demonstrated in bumble bees, including altered gene expression (Bebane et al. 2019; Colgan et al. 2019; Martín‐Blázquez et al. 2023; Witwicka, López‐Osorio, Arce, et al. 2025), reduction of foraging efficiency (Siviter et al. 2021; Stanley et al. 2016; Switzer and Combes 2016), impairment of learning and memory (Stanley et al. 2015), disruption of immune response (Czerwinski and Sadd 2017), reduction of queen hibernation success (Fauser et al. 2017) and several measures of colony success (Chole et al. 2022; Fauser‐Misslin et al. 2014; Leza et al. 2018; Stuligross and Williams 2020). It is plausible that bumble bee species are differentially exposed to neonicotinoid residues, depending on their foraging behaviour, but outside of research on feeding and ovary development (Baron et al. 2017), we know little about species‐specific variation in responses to controlled exposures.

In this study, we compare larval gene expression responses of two declining wild bumble bee species in the subgenus *Bombus s. s*. ( *B. occidentalis* and *B. terricola* ) and two wild stable species in different subgenera, *B. impatiens* (*Pyrobombus*) and *B. griseocollis* (*Cullumanobombus*) exposed to the pathogen *N. bombi*, the neonicotinoid pesticide imidacloprid and the combination of the two. Larval health is critical for colony success and this developmental stage may be particularly sensitive to environmental stressors. Previous research has demonstrated that larvae of *B. impatiens* exhibit an acute transcriptomic response (Martín‐Blázquez et al. 2023) and harmful effects on the nervous system (Smith et al. 2020) when exposed to imidacloprid, and infection with *N. bombi* is considered to occur primarily from larval exposure (Calhoun et al. 2021; Rutrecht et al. 2007). We examine the hypothesis that bumble bees undergo species‐specific molecular responses to the exposure of pathogen and pesticide stressors, and more specifically, that declining and stable species have different responses that may explain differences in their population health status and reported differences in infection (Cameron et al. 2011; Cameron et al. 2016; Gillespie 2010). We predict that the degree of gene expression within gene pathways involved in resistance or tolerance to the respective stressors, such as immune (Barribeau et al. 2015) and detoxification (Tsvetkov et al. 2021) pathways, will vary among species, depending on the health of their populations (declining or stable).

Initially, we employed RNA‐seq analysis to compare whole transcriptome expression profiles across treatments and species for *B. occidentalis* and *B. impatiens* larvae. We subsequently sought to expand our understanding of differences among species by targeted expression analysis with quantitative PCR. In this experiment, we include *B. griseocollis* as an additional independent subgenus representative of a stable species and the declining *B. terricola* to examine whether differential responses in *B. occidentalis* are shared with other declining species in the subgenus *Bombus s. s*. (Cameron et al. 2011). In all four species, we examined the expression of a subset of genes (*n* = 20) selected from the genome‐wide expression analysis of responses to the individual and combined stressor treatments. This approach enabled us to identify species‐specific molecular responses of bumble bees to these stressors and to assess any association with their known population status. This conservation‐orientated research includes vulnerable species and rearing logistics that constrain large colony sample sizes. Nonetheless, our approach yields realistic field‐approximated data for examining wild bee health relative to the use of single‐species approaches with commercially raised colonies. Results from colonies grown from wild collected spring queens in place of species that have been captively bred for commercial pollination over decades, such as *B. impatiens* colonies used in many studies on responses to stressors (Cameron and Sadd 2020), are more directly relevant to the conservation of wild bees. We also restricted our use of colonies to those that were free of identifiable common pathogens, thus constraining the number of *B. terricola* and *B. occidentalis* colonies available for experiments due to their high infection prevalences in the field (described in Cameron et al. 2011, 2016). This, however, ensures our results are not confounded by existing infections.

## 2 Methods

### 2.1 Bumble Bee Source Colonies

Experimental larval bees were derived from laboratory colonies reared from wild‐caught gynes of *B. impatiens* (*n* = 4 colonies), *B. occidentalis* (*n* = 4) and *B. terricola* (*n* = 2) in 2019 and *B. impatiens* (*n* = 3) and *B. griseocollis* (*n* = 4) in 2020. The four chosen taxa represented both stable species of least concern ( *B. impatiens* and *B. griseocollis* ) and declining species deemed vulnerable ( *B. occidentalis* and *B. terricola* (https://www.iucnredlist.org)). *Bombus impatiens* and *B. griseocollis* are abundantly distributed in central and eastern North America, and their populations are stable (Cameron et al. 2011) and even expanding, in the case of *B. impatiens* (Looney et al. 2019). *Bombus occidentalis* is restricted to western North America and has undergone precipitous population declines across its range over the last several decades (Cameron et al. 2011; Janousek et al. 2023); *B. terricola* is found in northeastern North America and has also experienced sharp declines in range and abundance (Cameron et al. 2011). *Bombus impatiens* and *B. griseocollis* gynes were collected from the Mackinaw River watershed (Lexington, IL, USA) with the permission of the ParkLands Foundation (http://www.parklandsfoundation.org). *Bombus terricola* queens were collected from northeast Vermont under the Vermont Agency of Natural Resources permit #ER‐2019‐10. *Bombus occidentalis* were collected from central Oregon and initially maintained for colony foundation (following Rowe et al. 2023) at the USDA‐ARS Pollinating Insect‐Biology, Management, Systematics Research Unit (Logan, UT, USA) until transferred to Illinois State University when first‐brood workers were produced. Upon receipt at ISU, all colonies were maintained under standard rearing conditions (Calhoun et al. 2021). Briefly, bees were kept at 26°C± 1.5°C under red light illumination and provided with honey bee‐collected pollen (Brushy Mountain Bee Farm, NC, USA) three times a week and sugar water (1 g cane sugar: 1 mL boiled tap water with 0.1% cream of tartar) *ad libitum*. Following the establishment of microcolonies (explained below), the pollen provided was honey bee‐collected pollen from CC Pollen Co. (https://www.beepollen.com, Phoenix, AZ, USA), which is collected in high desert habitat away from agricultural or residential areas and determined to be pesticide‐free (McArt et al. 2017). Colonies were confirmed free of common pathogens (e.g., *Nosema bombi* and *Crithidia bombi*) by obtaining and observing faecal samples under phase contrast microscopy (400 × total magnification) from a subset of workers and the queen. This was done when colonies had 4–8 workers and again prior to the establishment of microcolonies.

### 2.2 Microcolony Design

Individual colonies derived from wild‐caught gynes served as source colonies for the establishment of microcolonies, defined here as small, queenless groups of bumble bee workers and a small portion of brood taken from the same parent colony (Martín‐Blázquez et al. 2023). Microcolonies provide standardised experimental units that are easy to manipulate experimentally. Briefly, each microcolony was established with three workers and a larval brood clump containing four to eighteen (mean ± SE = 6.38 ± 0.44) size‐controlled worker larvae. A larval size that approximated third instar larvae was chosen, with larval size/instar determined by visual comparison with other instars of smaller (younger first and second instar larvae) or larger (older prepupal larvae) sizes. From each source colony, four microcolonies were established per experimental replicate, resulting in a total of 28 microcolonies (from 7 source colonies) for *B. impatiens* , 16 microcolonies (4 colonies) for *B. occidentalis* , 8 microcolonies (2 colonies) for *B. terricola* and 16 microcolonies (4 colonies) for *B. griseocollis* over the two years.

Microcolonies were provisioned initially with standard sugar water and pollen dough *ad libitum* to allow them to acclimate for 24 h. Sugar water was prepared as above for maintaining source colonies, and this same sugar water solution was used to prepare pollen dough by mixing with ground honey bee‐collected pollen from CC Pollen Co. at a v/w ratio of 1:3.2.

### 2.3 Imidacloprid and *Nosema bombi* Exposure Treatments

After 24‐h acclimation, larvae from each replicate microcolony from a source colony (4 microcolonies per source colony) were exposed over a precise time schedule to one of the following four treatments: (i) unexposed to imidacloprid or *N. bombi* (control), (ii) exposed to *N. bombi* only, (iii) exposed to imidacloprid only or (iv) exposed to both imidacloprid and *N. bombi*. Imidacloprid and *N. bombi*‐treated diets were prepared fresh before each experimental exposure to ensure that imidacloprid was not degraded and *N. bombi* was viable. Imidacloprid was prepared at 7 ppb concentration in both sugar water and pollen dough, while unexposed controls (0 ppb) received similarly treated but unspiked provisions, as described in Martín‐Blázquez et al. (2023). The imidacloprid concentration used represents field realistic ranges and has been shown to lead to differences in gene expression between exposed and unexposed *B. impatiens* larvae (Martín‐Blázquez et al. 2023).

After the 24‐h acclimation, two microcolonies from each source colony were given unspiked sugar water and pollen (treatments i and ii above) and two were given imidacloprid‐treated provisions (treatments iii and iv above). The imidacloprid diet treatments were given for 72 h. At the cessation of the pesticide treatment, each larva in the microcolonies received either a control inoculum without spores (treatments i and iii above) or a 2 μL inoculum of the *N. bombi* solution (20,000 spores total) to consume (treatments ii and iv above). An inoculation solution of *N. bombi* spores (isolate O17.01) at 10,000 spores per μL in a sugar water and pollen mix was prepared as described in (Calhoun et al. 2021). Briefly, prepared spore aliquots stored at −80°C were thawed and spore concentration quantified. Solutions were centrifuged at 3000 g for 5 min, the supernatant removed and the spore pellet resuspended in a 1:1 v/v combination of ultrapure water and sugar water blended with five grains of fresh pollen. The volume of this blended food was adjusted based on the starting spore concentration to achieve a spore solution of 10,000 spores per μL. Individual larvae received either this solution or a comparable spore‐free control solution. Larval brood were temporarily removed from their microcolony, the wax covering was carefully peeled back, and the inoculum was delivered to the ventral side of each individual using a micropipette. The brood were placed back into their respective microcolony following observed consumption of the inoculum.

At 24 h after *N. bombi* treatment (see Liu et al. 2020 and Yue et al. 2015 for significance of 24‐h timepoint after larval infection with Microsporidia), four larvae from each of the four treatment microcolonies were collected in 2 mL cryotubes, flash frozen with liquid nitrogen and stored at −80°C until they were processed. We were interested in early differential molecular responses of the hosts to pathogen exposure, which can result in different infection outcomes. At the timepoint that the treated larvae were collected, it was not possible to verify infection. However, the exact spore source and protocol have been documented to lead to infections in *B. impatiens* (Calhoun et al. 2021; Martinez et al. 2023).

### 2.4 RNA Extraction and RNA‐Seq Analysis

We extracted RNA from three frozen larvae per microcolony for all treatments of each species with the E.Z.N.A. Total RNA Kit I (Omega Bio‐tek, Georgia, USA) following the manufacturer's instructions, including a standard treatment with DNAse I. We checked total RNA integrity and quantity by running 1 μL of RNA on a 1.0% agarose gel and by analysing 1 μL of total RNA with a Qubit fluorometer (Thermo Fisher Scientific). In our first experiment, we conducted RNA‐seq analysis on *B. impatiens* and *B. occidentalis* . We pooled a portion of the three individually‐collected, quality‐checked larval RNA samples from each of the 28 *B. impatiens* and 16 *B. occidentalis* microcolonies, to obtain a final concentration of 1 μg RNA per pooled sample. An outlier *B. occidentalis* colony was later removed from analysis (SI Methods). Pooled RNA samples from a microcolony were treated with poly‐A tail selection and sequenced using Illumina NovaSeq6000 sequencer at W.M. Keck Center for Comparative and Functional Genomics, Roy J. Carver Biotechnology Center, University of Illinois Urbana‐Champaign, USA.

Illumina sequencing produced an average ± SD of 37.0 ± 8.1 million reads per library for *B. impatiens* and 31.8 ± 4.3 million reads per library for *B. occidentalis* (raw reads are available in the SRA repository (NCBI) under the BioProject PRJNA1071869). All reads were 100 bp with a Phred score greater than 35 per nucleotide position. We trimmed adapter sequences and low‐quality bases (trimming criteria in SI) from the reads with Trimmomatic v0.38 (Bolger et al. 2014). FastQC (Andrews 2010) was used to visualise the quality check of the reads before and after the trimming (Table S1). We mapped trimmed reads of *B. impatiens* and *B. occidentalis* to the *B. impatiens* genome v2.2 (Sadd et al. 2015) (GenBank accession number GCF_000188095.3) with STAR v2.7 (Dobin et al. 2012), with *B. occidentalis* and *B. impatiens* mapping rates being 88.7% [88.0%–90.1%] and 95.2% [94.3%–96.6%], respectively. Mapping the reads to the *B. terrestris* genome (Sadd et al. 2015) (GenBank accession GCA_000214255.1) was also examined; however, the *B. impatiens* genome v2.2 alignment was chosen because of the more complete annotation status of its reference genome. Further mapping and reference genome choice details are provided in SI Methods (Figure S1 and Table S1). We summarised the read counts from gene features with *htseq‐count* using the ‘union’ method (Anders et al. 2014).

We performed two complementary approaches to analyse the whole genome transcriptomic responses of the two species to the stressor exposure treatments: differential gene expression analysis and a gene co‐expression network analysis. Differential gene expression analysis identifies individual genes, offering a straightforward way to highlight potentially relevant biomarker genes for the responses to our stressor treatments and how those may differ across the two compared species. The second approach of building a network of co‐expressed genes using weighted gene co‐expression network analysis (*WGCNA*) allows us to uncover modules of genes that are co‐expressed across samples, likely reflecting shared regulation or participation in the same biological processes. This systems‐level approach reveals coordinated expression patterns that may not be apparent from differential gene expression alone.

#### 2.4.1 Testing for Differential Gene Expression

After employing the relative log expression normalisation (RLE) method, we tested for differential expression with *DESeq2* (Love et al. 2014) for *B. occidentalis* and *B. impatiens* separately, including combined imidacloprid and *N. bombi* treatment and colony as factors (SI Methods; Figure S2). This accounted for between colony variation, but the interaction between colony and treatment could not be included in the model as our design includes just one replicate microcolony per treatment per original source colony. Given the differences in sample number, we tested whether the number of differentially expressed genes (DEG) between *B. occidentalis* and *B. impatiens* might be affected by sample number by means of an iterative resampling approach (SI Methods). After repeating the *DESeq2* analysis with several combinations of sample subsets from *B. impatiens*, we determined there were no significant differences between the subsets and the whole‐sample analyses (Table S2) (hypergeometric test: *p* < 0.001). Furthermore, after exploring preliminary results, we found that *B. occidentalis* samples from colony 19.235 were outliers across all treatments, despite high‐quality sequences, and thus removed them from further analysis (SI Methods; Table S3 and Figure S3). We considered a gene to be differentially expressed if its false discovery rate (FDR) was less than 0.05 in at least one comparison between the control and any of the three treatments. We used a hypergeometric test to check if the number of overlapping genes from two lists of DEGs was higher than expected by chance.

#### 2.4.2 Gene Co‐Expression Network Analysis

We built a network of co‐expressed genes with weighted gene co‐expression network analysis (*WGCNA*) (R package (Langfelder and Horvath 2008)) using the gene expression data from 12,736 genes expressed in all samples combined (28 from *B. impatiens* and 12 from *B. occidentalis* ) as input. After building an unsigned network with a soft threshold value of eight (recommended for unsigned networks built with 30–40 samples (Langfelder and Horvath 2008)), modules of co‐expressed genes were obtained using a hybrid tree cutting method (additional *WGCNA* parameters in SI Methods). The effects of species, treatment and colony on each module were tested with a Kruskal–Wallis test followed by post hoc Dunn's tests. In addition, we determined the most connected genes (top hub genes) from each module.

### 2.5 Gene Ontology (GO) Term Enrichment Analysis

We retrieved the GO terms from the *B. impatiens* v2.2 genome gene set from the Hymenoptera Genome Database (Walsh et al. 2022) (accessed 30 November 2021). We used *topGO* (Alexa et al. 2006) to test for GO term enrichment in the up and downregulated DEG sets affected by each treatment, as well as for lists of genes included in each *WGCNA* module. We restricted this to the biological process ontology. A GO term was considered enriched if the weighted Fisher's exact test *p* value, corrected through FDR, was less than 0.05.

### 2.6 Quantitative Polymerase Chain Reaction (qPCR) Analysis

The above differential gene expression analysis from the RNA‐seq experiment was used to inform the subsequent targeted qPCR experiment that included additional samples from *B. griseocollis* and *B. terricola* . We carried out qPCR on each individual RNA sample extracted from single larvae from all treated microcolonies of *B. impatiens* , *B. occidentalis* , *B. terricola* and *B. griseocollis* . This was done to validate the gene expression patterns from 20 DEGs of interest selected from the *B. impatiens* and *B. occidentalis* RNA‐seq analysis (the same RNA samples used for RNA‐seq were tested, but run as unpooled individual samples), and to test how consistent the gene expression differences were in the other two species. The selected 20 DEGs for qPCR from the RNA‐seq experiment were differentially expressed in at least one comparison in one species, and were associated with at least one GO term enriched in any DEG list or with a stress response GO term, such as innate immune response (GO:0045087) or response to oxidative stress (GO:0006979) (Table S4). We designed primers for these target genes with PrimerBLAST (NCBI) using available versions of *B. terrestris* (Sadd et al. 2015) (GenBank accession GCA_000214255.1), *B. impatiens* (Sadd et al. 2015) (GenBank accession GCF_000188095.3), *B. terricola* (Kent et al. 2018) (SRA accession SRX3517961) and *B. cullumanus* (Sun et al. 2020) (GenBank accession GCA_014737535.1) genomes as sequence templates. Some primers were used for more than one species (Table S4). We synthesised cDNA with the iScript cDNA kit (BioRad) from 1 μg of total RNA per sample. We used 25 ng of cDNA per qPCR reaction following the Luna qPCR reaction kit (New England Biolabs, Massachusetts, USA) manufacturer instructions. We set the qPCR conditions in a StepOne Thermocycler (Thermo Fisher Scientific, Massachusetts, USA) as follows: hot start at 95°C for 1 min, then 40 cycles at 95°C for 15 s and 60°C for 30 s each, and finally a melt curve ranging from 60°C to 95°C with reads occurring every 0.3°C. We first tested the stability of expression for four housekeeping genes studied in *B. terrestris* (Horňáková et al. 2010) and analysed them with GeNorm v3 software (Vandesompele et al. 2002). We calculated relative quantities (RQs) following the Pfaffl method (Pfaffl 2001).

To calculate differences in expression between treatments for target genes in the qPCR data, we used a Kruskal–Wallis rank sum test and applied a Dunn's test post hoc analysis when testing pair‐wise treatment comparisons. Data from qPCR and RNA‐seq of *B. occidentalis* and *B. impatiens* were compared through correlation analysis to check the adjustment between techniques (SI Methods). We standardised the logFC (RQs) by transforming them into Z‐scores with mean = 0 and standard deviation = 1, then used hierarchical clustering to generate a dendrogram (based in Euclidean distance) to group all the qPCR results by similarities in expression. We performed these analyses in R v4.1.2 (R Core Team 2021).

## 3 Results

### 3.1 RNA‐Seq Gene Expression Responses Following Independent *N. bombi* and Imidacloprid Treatments

**FIGURE 1:**
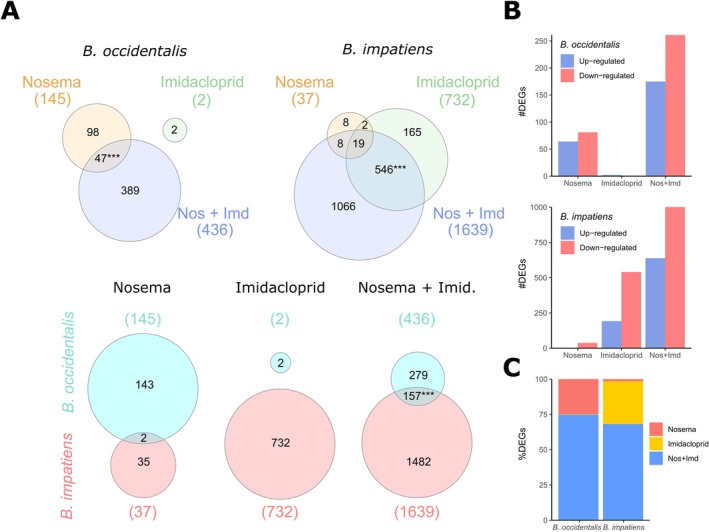
Differential expression analysis results. (A) Venn diagrams with the differentially expressed genes, showing intraspecific comparisons (top) and interspecific comparisons (bottom). Each coloured circle represents a treatment (top) or a species (bottom). Coloured numbers in parenthesis are the total number of differentially expressed genes (DEGs) in each comparison. Numbers inside circles indicate the number of DEGs for each category, asterisks represent statistical significance of hypergeometric tests (*** = *p* < 0.001). (B) Number of DEGs per species and treatment, showing upregulated (blue bars) and downregulated genes (red bars). (C) Percentage of DEGs per species for *Nosema bombi* (red portion), imidacloprid (yellow portion) and *N. bombi* plus imidacloprid (blue portion) treatments.

**FIGURE 2:**
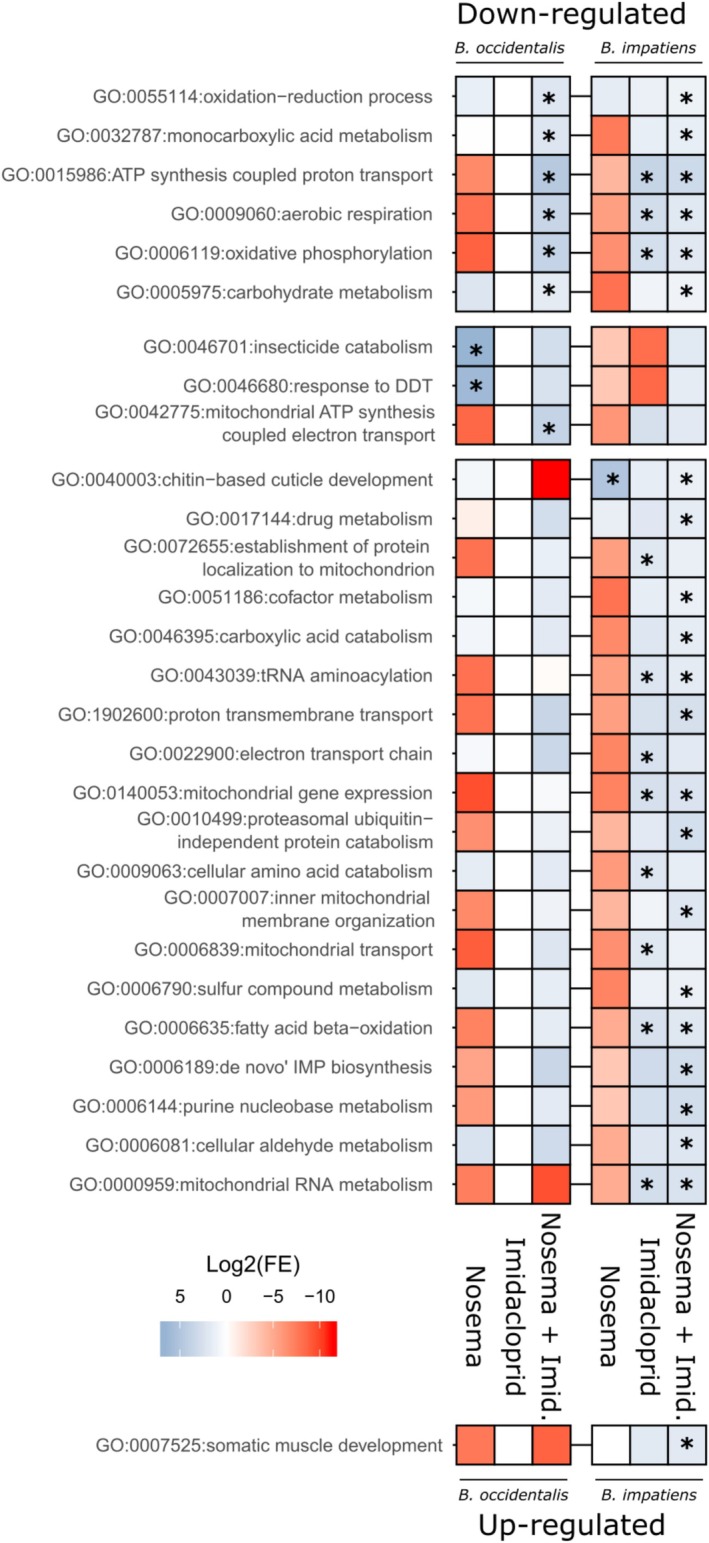
GO term enrichment analysis results from differentially expressed genes. Each tile represents enriched GO terms from differentially expressed gene (DEG) lists (top heatmap: Downregulated DEGs, bottom heatmap: Upregulated DEGs) per species and treatment, for *B. occidentalis* (left) and *B. impatiens* (right) DEG lists. Rows show the GO terms enriched in at least one DEG list. Columns show the treatments, grouped by species. Tiles are coloured in accordance with the logarithm to the base two of the fold enrichment of each term, indicating either enrichment (blue) or depletion (red) of the GO term in a DEG list. An asterisk indicates the GO term was enriched in the analysed gene list (FDR < 0.05).

Following separate exposures to *N. bombi* and imidacloprid, we found divergent differentially expressed gene profiles between larvae of *B. occidentalis* and *B. impatiens* (Figures 1, S4, S5 and S6; Tables S5 and S6). *Nosema bombi* exposure in *B. occidentalis* resulted in a larger number of differentially expressed genes, both upregulated and downregulated (145 DEGs: 64 upregulated, 81 downregulated), relative to *B. impatiens* (37 DEGs, all downregulated). Only two (5%) of the *B. impatiens* DEGs were present in the *B. occidentalis* DEG list. The top DEGs under *N. bombi* exposure in *B. occidentalis* included downregulation of *cytochrome P450 6 k1* (LOC100744499) and *CB1 cannabinoid receptor‐interacting protein 1‐like* (LOC100745470) (all LOC IDs are from the *B. impatiens* genome v.2.2). We found two enriched GO terms in the *B. occidentalis* downregulated DEGs following *N. bombi* exposure: insecticide catabolism (GO:0046701) and response to DDT (GO:0046680) (Figure 2 and Table S7). In *B. impatiens* , top DEGs under *N. bombi* exposure included downregulation of *mediator of RNA polymerase II transcription subunit 15‐like* (LOC105680379) and *larval cuticle protein A2B‐like* (LOC100745774). The sole enriched GO term for the *B. impatiens* DEG set downregulated by *N. bombi* was chitin‐based cuticle development (GO:0040003) (Figures 2 and S8).

The species‐biased pattern of differential gene expression seen in larvae under *N. bombi* exposure was reversed for imidacloprid exposure relative to the control group, with only two differentially upregulated genes in *B. occidentalis* (two uncharacterised proteins LOC100744115 and LOC100747998) versus 732 (192 upregulated and 540 downregulated) in *B. impatiens* , with no overlap of these differential gene expression sets. The top DEGs in *B. impatiens* included downregulation of the *mediator of RNA polymerase II transcription subunit 15‐like* (LOC105680379) again and, additionally, *CB1 cannabinoid receptor‐interacting protein 1‐like* (LOC100745470). In the *B. impatiens* gene set downregulated upon imidacloprid exposure, enriched GO terms were related to mitochondrial activity, including oxidative phosphorylation (GO:0006119), mitochondrial gene expression (GO:0140053) and establishment of protein localisation to mitochondrion (GO:0072655) (Figure 2 and Table S8). In *B. occidentalis* there was no overlap in DEGs from the separate *N. bombi* and imidacloprid exposures, but in *B. impatiens* 21 (56%) of the DEGs under the *N. bombi* treatment were also found following imidacloprid exposure.

### 3.2 RNA‐Seq Gene Expression Responses Following *N. bombi* and Imidacloprid Co‐Exposure Across Species

Relative to the magnitude of the different gene expression responses between species following single treatments, there was significantly greater similarity under the combined *N. bombi* plus imidacloprid treatment. Nonetheless, both species displayed unique differences (Figures 1, S4, S5 and S6; Tables S5 and S6). In the combined treatment relative to the control group, we found 436 DEGs (175 upregulated and 261 downregulated) in *B. occidentalis* and 1639 (638 upregulated and 1001 downregulated) in *B. impatiens*. A core set of 157 DEGs (36% of the *B. occidentalis* differential response) was shared (higher than expected by chance, hypergeometric test: *p* < 0.001).

In *B. occidentalis* , enriched GO terms of the downregulated DEGs under the combined treatment related to mitochondrial activity and cellular respiration, including oxidative phosphorylation (GO:0006119), aerobic respiration (GO:0009060) and mitochondrial ATP synthesis coupled electron transport (GO:0042775) (Figure 2 and Table S7). In *B. impatiens* , upregulated DEGs showed only one enriched GO term (somatic muscle development, GO:0007525) (Figure 2 and Table S8); the downregulated set included many of the already mentioned mitochondrial function‐related terms found in *B. occidentalis* , but also chitin‐based cuticle development (GO:0040003), as in the *B. impatiens* response to *N. bombi* exposure alone.

In the *B. occidentalis* DEG set following combined exposure, 47 (11%) of the DEGs were also found in the *N. bombi*‐only response, which is significantly higher than expected by chance (hypergeometric test: *p* < 0.001). The top DEGs in *B. occidentalis* included a downregulated *nucleoside diphosphate kinase* (LOC100743770) and an upregulated *prohormone‐2* (LOC100744531). Meanwhile, in the *B. impatiens* differentially expressed gene set, 365 (35%) of the DEGs were also found in the *B. impatiens* response to imidacloprid exposure, a significantly higher overlap than expected by chance (hypergeometric test: *p* < 0.001). As in the single exposure treatments, the *mediator of RNA polymerase II transcription subunit 15‐like* (LOC105680379) was downregulated along with the *allergen Cr‐PI‐like* (LOC100741500) in the top DEGs.

In addition to the effects of exposure treatment, there were also colony‐of‐origin effects in *B. occidentalis* (Table S9) and *B. impatiens* (Table S10).

### 3.3 Combined Co‐Expression Network Analysis of Species‐Specific Responses

**FIGURE 3:**
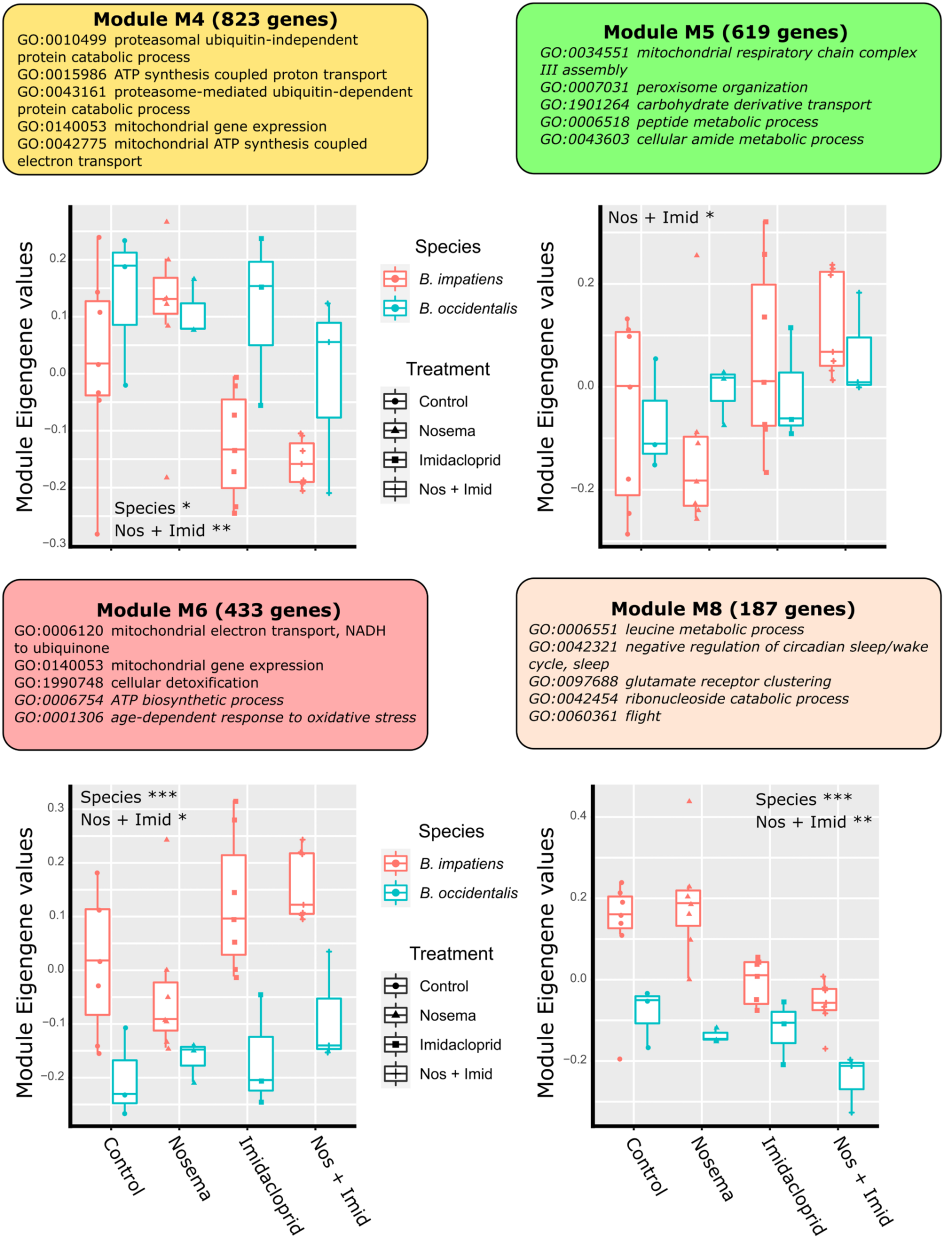
Co‐expressed gene modules with differences between control and treatments. Each plot shows the module eigengene (ME) values for the four modules that showed statistical differences between control and one of the treatments. Plot headers show the module name, the number of genes within the module in parentheses and the top five most enriched GO terms within the module (GO terms in italics indicate the enrichment was not statistically significant). X‐axis shows the treatments. Y‐axis shows the ME values for each treatment and species. The boxes are colour coded for species (red for *B. impatiens* , blue for *B. occidentalis* ). Individual sample's ME values are also plotted per treatment and species to show a more detailed view of the ME value distribution. Text in the graphic area indicates the significant differences between comparisons (species: Significance between *B. occidentalis* and *B. impatiens* ME values; Nos + Imid: Significance between control and *Nosema bombi* plus imidacloprid treatments). Asterisks indicate the level of statistical significance (* = 0.05 > *p* > 0.01, ** = 0.01 > *p* > 0.001, *** = *p* < 0.001).

The WGCNA grouped 7982 genes into 12 detected modules of co‐expressed genes (Tables S11, S12, S13 and Figure S7). Analysing identified co‐expressed gene modules, we found differences between the control and the combined treatment within four modules (Figure 3 and Table S14). There were interspecific differences within eight modules (Table S14). Focusing on the two modules (M4 and M6) with both significant treatment effects (Figure 3 and Table S14) and significantly enriched GO terms (Table S13), the observed patterns indicate species‐specific responses. Module M4 showed module eigengene (ME) values that were reduced with imidacloprid exposure and its combined exposure with *N. bombi*, but this pattern was more apparent in *B. impatiens* than *B. occidentalis*. Module M4 is significantly enriched for 11 GO terms related to proteasome assembly and cell respiration, linking its biological function to protein degradation and mitochondrial activity. Module M6 showed a strong species effect and an increase in ME values in imidacloprid exposure and the combined treatments relative to unexposed controls, with this effect again appearing more pronounced in *B. impatiens* than *B. occidentalis*. Module M6 is enriched for the GO terms mitochondrial electron transport (GO:0006120), mitochondrial gene expression (GO:0140053) and cellular detoxification (GO:1990748), again related to mitochondrial activity in addition to detoxification.

### 3.4 Targeted qPCR Gene Expression Response Across Four Bumble Bee Species to *N. bombi*, Imidacloprid and Their Combination

**FIGURE 4:**
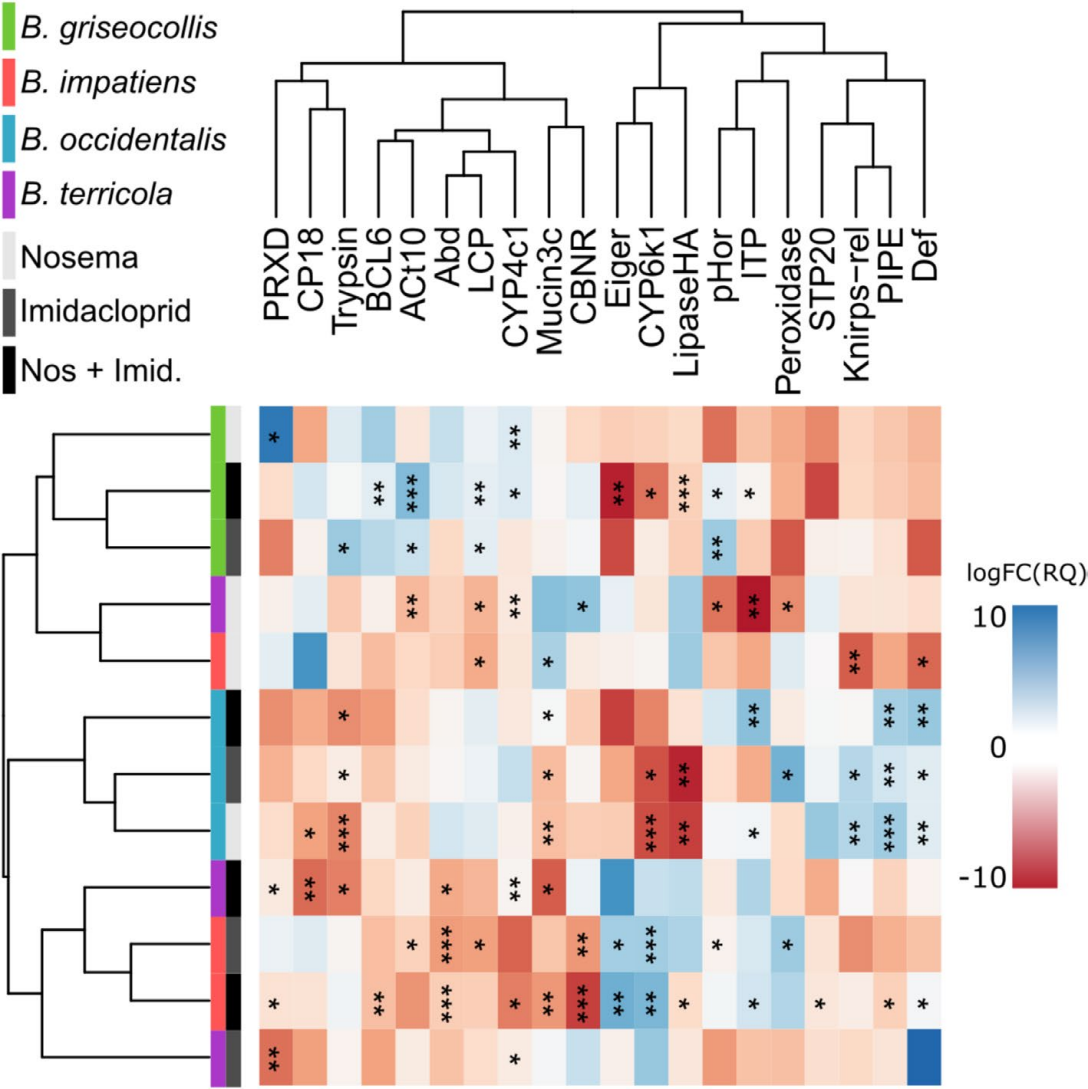
The relative quantity (RQ) of gene expression across twenty targeted genes of four bumble bee species exposed to *Nosema bombi* and imidacloprid treatment combinations compared to unexposed controls. Rows in the matrix represent the species and treatment combinations: The species are colour coded in the first column of the left dendrogram, while treatments are coded in grey scale in the second column of the left dendrogram. Columns represent genes assessed (Table S4). Dendrograms were generated with hierarchical clustering of the logarithm to the base two of the fold change of the RQ. Blue tiles represent upregulated expression compared to the control; red tiles represent downregulated expression compared to the control. * = 0.05 > *p* > 0.01, ** = 0.01 > *p* > 0.001, *** = *p* < 0.001.

Targeted qPCR on the 20 DEGs selected based on the RNA‐seq analysis was applied to *B. impatiens* and *B. griseocollis* , representing stable species and *B. occidentalis* and *B. terricola* , representing declining species. Results validated the RNA‐seq analysis. RNA‐seq expression levels from the pooled RNA samples from *B. impatiens* and *B. occidentalis* microcolonies were strongly positively correlated with the qPCR‐derived relative quantities from individual samples (Figure S8). The gene expression response profiles across the 20 targeted genes for the four species were diverse and clustered by species as often as by treatment (Figure 4). Hierarchical clustering based on the relative gene expression quantities within species grouped all experimental treatments of *B. occidentalis* and *B. griseocollis* into species‐specific clusters. The *N. bombi* treatment profiles of *B. impatiens* and *B. terricola* , however, formed a single cluster, while the imidacloprid and combined imidacloprid plus *N. bombi* responses formed another cluster, albeit with distinctness between the species expression profiles within these clusters.

## 4 Discussion

Our results reveal species‐specific molecular responses in bumble bees exposed to a neonicotinoid pesticide and a microsporidian pathogen, stressors frequently reported as potential drivers of decline. The declining *B. occidentalis* and stable *B. impatiens* have species‐specific genome‐wide gene expression patterns in larvae from microcolonies with imidacloprid‐spiked provisions and in larvae exposed to spores of *N. bombi*. Both species show a greater than expected number of overlapping DEGs when stressors are combined than when introduced singly but nevertheless display a higher number of species‐specific DEGs. Our targeted qPCR gene expression approach, adding a second declining ( *B. terricola* ) and stable ( *B. griseocollis* ) species, likewise indicates species‐specific variation in gene expression among the species across a selection of 20 chosen genes. Counter to our specific hypothesis that differential expression responses would be tied to species decline status, gene expression was not significantly correlated with species decline, nor was there a correlation with species relatedness (e.g., the two *Bombus s. s*. representatives were not more similar).

Recent studies have highlighted the ability of bumble bee transcriptomics to potentially detect exposure to stressors in landscape settings (Quinlan et al. 2025; Tsvetkov et al. 2021), including potential pathogen and pesticide exposure. However, such studies do not allow for causal links to be established between specific stressor exposures and responses. Our results allow us to pinpoint molecular responses to controlled, specific exposures to pesticide and pathogen stressors and their combination, and how they differ across species. The greater number of DEGs detected in *B. occidentalis* relative to *B. impatiens* in response to *N. bombi* spore exposure confirms species‐specific differences in response to pathogens. The *B. impatiens* response is not a subset of the *B. occidentalis* response, but rather the two species respond in different ways, with only 5% of the fewer DEGs in *B. impatiens* being shared with those of *B. occidentalis*. Our focus on the acute molecular response does not allow us to determine whether these expression differences ultimately affect infection outcomes but given the known host health‐ and fitness‐related impacts of *N. bombi* infection (Martinez et al. 2023; Otti and Schmid‐Hempel 2007, 2008; Rutrecht and Brown 2009), it is probable that differential molecular responses soon after exposure would have subsequent consequences. The greater gene expression response in *B. occidentalis* could indicate that this species is more impacted by *N. bombi* exposure. Conversely, *B. impatiens* appears relatively tolerant to *N. bombi* infection (Calhoun et al. 2021). Importantly, *N. bombi* exposure in *B. occidentalis* results in a DEG set enriched for downregulation of genes with GO terms associated with the processing of insecticides, which was not the case for *B. impatiens* . This could offer a molecular angle for the multiple stressor hypothesis (Botías et al. 2021; Calhoun et al. 2021; Straub et al. 2023), with pathogen exposure compromising a host's response to pesticides. While our spore source and exposure protocol has resulted in *B. impatiens* infections in prior studies (Calhoun et al. 2021; Martinez et al. 2023), we do not verify larval infection of either species in this study as our design was to analyse acute molecular responses in larvae with *N. bombi* exposure. The differential transcriptomic responses reported here nonetheless expand the knowledge of species‐specific outcomes of *N. bombi* exposure at the molecular level. They could potentially provide an underlying context for previously demonstrated differences in the effects of *N. bombi* infection on colony demographics of two European bumble bee species, *B. terrestris* and *B. lucorum* (Rutrecht and Brown 2009).

Evidence for a genome‐wide species‐specific response is not only present with pathogen exposure, which might be expected given the dynamic evolutionary relationship between hosts and pathogens, but also with pesticide exposure to imidacloprid. While we see an extensive response of *B. impatiens* to imidacloprid, *B. occidentalis* exhibits an extremely limited response, with no overlap between the two species. The downregulated gene set of *B. impatiens* includes an enrichment of GO terms associated with mitochondrial activity and gene expression, mirroring previous findings in larvae under the same exposure protocol (Martín‐Blázquez et al. 2023). Moreover, the gene co‐expression network analysis further underlines these differences, particularly within modules M4 and M6, relating to mitochondrial activity and detoxification, which are more responsive to the imidacloprid and combined treatments in *B. impatiens* than in *B. occidentalis*. Such species‐specific responses could lead to different outcomes between species for both individual and colony level traits known to be affected by neonicotinoid exposure (Chole et al. 2022; Czerwinski and Sadd 2017; Fauser et al. 2017; Fauser‐Misslin et al. 2014; Leza et al. 2018; Siviter et al. 2021; Stanley et al. 2015; Stanley et al. 2016; Stuligross and Williams 2020; Switzer and Combes 2016), resulting in divergent population health effects in environments with widespread and persistent neonicotinoid use. Although comprising different specific genes, a clear molecular response in *B. impatiens* to neonicotinoid exposure aligns with findings from other insects, including the bumble bee *B. terrestris* (Colgan et al. 2019; Witwicka, López‐Osorio, Arce, et al. 2025; Witwicka, López‐Osorio, Chaudhry‐Phipps, and Wurm 2025), honey bees (Fent et al. 2020; Tsvetkov and Zayed 2021) and *Drosophila* (Martelli et al. 2023). Similarities include effects of exposure on genes involved in energy homeostasis and detoxification. These same responses were not, however, shared by *B. occidentalis* . Although the distinct molecular responses between our investigated species could lead to previously reported species‐specific outcomes in individual‐level phenotypic traits following neonicotinoid exposure (Baron et al. 2017), further studies would elucidate direct links between molecular and individual as well as colony and population health levels.

The molecular responses of *B. occidentalis* and *B. impatiens* larvae were more aligned in the combined treatment, with 36% of the DEGs being shared relative to the controls. Nonetheless, they showed considerable variation in response. Importantly, comparison of the DEG sets of the individual stressors to those of the combined suggests that responses to individual stressors are not simply additive when encountered simultaneously. Indeed, other evidence suggests that a potentially advantageous response to an individual stressor may be compromised when stressors are encountered simultaneously. For example, it was shown elsewhere that in *B. impatiens* the GO term *chitin‐based cuticle development* is enriched in upregulated genes in response to imidacloprid (Martín‐Blázquez et al. 2023), but in the *N. bombi* and combined treatments in the current study, the same GO term was significantly enriched in the down‐regulated gene set of *B. impatiens* , suggesting a shift of the response to imidacloprid when exposed simultaneously to *N. bombi*.

Species‐specific responses to stressors may underlie the heterogeneous patterns of population health among North American bumble bee species in recent decades (Cameron et al. 2011). We provide strong overall support for this from both the transcriptomes and the gene co‐expression network analysis of the declining *B. occidentalis* and stable *B. impatiens* . The response to *N. bombi* exposure is particularly interesting, given the significant correlation found between species decline status and *N. bombi* prevalence (Cameron et al. 2011; Cameron et al. 2016). Our targeted qPCR data across four species from 20 targeted genes, despite uncovering further evidence for species‐specific variation in genomic responses, do not, however, support the hypothesis that species population decline in *Bombus* is linked to the gene expression responses measured here. Neither the declining nor the stable species grouped together by similarity in expression patterns. Instead, *B. impatiens* (stable) and *B. terricola* (declining) cluster in their responses to *N. bombi*, imidacloprid and combined treatments, albeit still exhibiting differences in the quantitative responses of individual genes. The clustering of these two species contrasts not only with their health status but also their phylogenetic relatedness, as *B. terricola* and *B. occidentalis* are instead closest relatives (Cameron et al. 2007). *Bombus occidentalis* (declining) and *B. griseocollis* (stable) are distantly related and exhibit distinct responses, attributable to species over treatment effects. Although our results do not support the hypothesis that the molecular responses of bumble bees to stressors are linked to their population health status (and phylogeny), further investigation of the hypothesis is warranted. Constraints to our investigation include focusing on a subset of 20 genes in the four‐species comparison based on the transcriptomes of only two species, which may have missed common patterns in other genes that would link species of similar decline status. Furthermore, molecular responses are temporally dynamic, and the time point chosen in this study to take a snapshot of the response may not be representative of the clustering of responses across species at other time points. There is also the potential for colony‐specific differences to mask the ability to resolve broader scale similarities such as relatedness and population health. In fact, we uncover colony‐level differences in responses in the transcriptomes of *B. occidentalis* and *B. impatiens* . If colony‐level variation is strong, then greater sample sizes will be needed to uncover more generalisable patterns. Due to conservation and logistical constraints on collecting and rearing colonies of vulnerable, uninfected species, sample sizes are likely to be limited. While this presents a difficulty for such studies, working with wild species, both vulnerable and stable, places the research into a more real‐world methodological framework for examining causal factors of species declines.

Our study focuses on two principal stressors. Yet, multiple factors likely threaten bumble bee individual, colony and population health, endangering these key pollinators (Cameron and Sadd 2020). We still know too little about how different bumble bee species respond to environmental stressors, especially on a molecular level, and whether differences in responses could explain divergent outcomes in shifting range and abundance of populations. Our results strongly suggest species‐specific molecular responses, however, to *N. bombi* and imidacloprid. We feel it important to point out that these results support prior assertions (Cameron and Sadd 2020) that caution is necessary when extrapolating conservation‐relevant findings from studies of only one or two commonly available domesticated species ( *B. impatiens* in North America and *B. terrestris* in Europe). Many other bumble bee species listed as least concern have disparate evolutionary histories and ecology and deserve further research into factors leading to their environmental resilience. Such species will provide key insights into the adaptive capacity of bumble bees in a world undergoing global biodiversity declines from human‐induced environmental stressors.

## Author Contributions

S.A.C. and B.M.S. designed the study. A.C.C., B.M.S. and J.P.S. reared colonies from field‐collected gynes. A.C.C. and R.M.B. conducted larval experiments. R.M.B. performed larval sample preparation, RNA extraction and bioinformatics. S.A.C., B.M.S. and R.M.B. wrote the manuscript. All authors contributed to its revision.

## Conflicts of Interest

The authors declare no conflicts of interest.

## Supporting information


FIGURES S1‐S8.

Table S1.

Table S2.

Table S3.

Table S4.

Table S5.

Table S6.

Table S7.

Table S8.

Table S9.

Table S10.

Table S11.

Table S12.

Table S13.

Table S14.

## Data Availability

Sequence data are available from NCBI sequence read archive (SRA) Bio Project PRJNA1071869 (https://dataview.ncbi.nlm.nih.gov/object/PRJNA1071869), under accession numbers SRR27842857 to SRR27842900. Experimental data (raw RNA‐seq counts and relative quantities from qPCR) and scripts are available from Dryad (https://datadryad.org/dataset/doi:10.5061/dryad.rxwdbrvjv).
