# Supplementary material for: Pesticide and Pathogen Exposure Causes Idiosyncratic Gene Expression Responses Across Four Diverse North American Bumble Bee Species
Source: Mol Ecol. 2025 Aug 8;34(17):e70042. doi: 10.1111/mec.70042 (PMC12376964; doi:10.1111/mec.70042)
Supplement: Supplementary file 1 — FIGURES S1‐S8. [file MEC-34-e70042-s013.pdf]

Supplemental Information for:

Supporting Information: Pesticide and pathogen exposure causes idiosyncratic gene expression responses in four North American bumble bee species

Rubén Martín-Blázquez[1], Sydney A Cameron[1], Austin C Calhoun[2], James P Strange[3], Ben M Sadd*[2]

[1] Department of Entomology, University of Illinois Urbana-Champaign, Urbana, IL 61801, USA

[2] School of Biological Sciences, Illinois State University, Normal, IL 61790, USA

[3] Department of Entomology, The Ohio State University, Columbus, OH 43210, USA

* Correspondence: bmsadd@ilstu.edu

Table of Contents:

**RNA-seq differential gene expression analysis pipeline: extended specifications**	Page 3
**Detecting noise in expression results for *B. occidentalis* data**	Page 5
**Testing sample size effect for *B. impatiens* dataset**	Page 5
**RNA-seq weighted gene co-expression network analysis pipeline: extended specifications**	Page 6
**Quantitative Polymerase Chain Reaction (qPCR) further specifications**	Page 7
**Supporting Information References**	Page 10
**Supporting Information Table Index**	Page 13
**Supporting Information Figures**	Page 14

RNA-seq differential gene expression analysis pipeline: extended specifications

Prior to differential gene expression analysis, we explored alternative approaches in our alignment and normalization of expression values. We compared aligned reads to the *B. impatiens* genome for both species and aligned *B. impatiens* reads to *B. impatiens* genome and *B. occidentalis* reads to *B. terrestris* genome. Because *B. occidentalis* is more closely related to *B. terrestris*, it was logical to expect better alignment results when mapping *B. occidentalis* RNA reads to the *B. terrestris* genome. However, the *B. impatiens* genome annotation is currently more advanced than the *B. terrestris* genome, which can provide more accurate information about gene expression.

Before aligning the reads to the genomes, we trimmed the reads using Trimmomatic v0.38 (Bolger *et al.*, 2014), using the following parameters: "*-phred33 ILLUMINACLIP:$trimm:2:30:10LEADING:28 TRAILING:28 SLIDINGWINDOW:4:15 MINLEN:30*". Trimming removed less than 1% of the nucleotides from all read samples due to the high quality of the sequencing. The trimmed reads were aligned to either the *B. impatiens* genome v2.2 or to the *B. terrestris* genome v1.0 (Sadd *et al.*, 2015) with STAR v2.7 (Dobin *et al.*, 2012), using the following parameters: "*--sjdbOverhang 99 --outSAMtype BAM SortedByCoordinate --quantMode GeneCounts --twopassMode Basic*". FastQC (Andrews, 2010) was used to count the total reads per RNA-seq library, before and after the trimming, and to summarize the percentage of aligned reads to genome from auxiliary files generated by STAR. The alignment rate was 95.21% [94.60% - 95.90%] for *B. impatiens* reads aligned to *B. impatiens* genome, 88.71% [88.00% - 90.10%] for *B. occidentalis* reads aligned to *B. impatiens* genome, and 95.50% [94.90% - 96.00%] for *B. occidentalis* reads aligned to *B. terrestris* genome. After alignment, read counts were summarized with *htseq-count* (Anders *et al.*, 2014), using

the following parameters: "*-q -f bam -t exon -i gene -m union -s no*". After establishing orthologous relationships between *B. impatiens* and *B. terrestris* proteins using BLASTp (Altschul *et al.*, 1990; Camacho *et al.*, 2009), PCA revealed almost no difference between the alignment approaches (Fig. S1). We thus selected *B. impatiens* genome as the reference to align the reads from both species to take advantage from its up-to-date annotation status.

We also compared the TMM normalization by *edgeR* and the custom normalization method from *DESeq2* before testing for differential expression. Once we had the read counts from HTSeq-count, we filtered the genes whose median expression was less than 1. To test differential expression with *DESeq2* (Love *et al.*, 2014) and *edgeR* (Robinson *et al.*, 2009), we designed an experiment matrix with the formula "~*treatment* + *colony*", where the factor *treatment* had four levels (control, imidacloprid, *Nosema* and combined) and the factor *colony* had as many levels as colonies in the experiment. We normalized the transformed counts using the trimmed median-means (TMM) method for *edgeR* and the relative log expression normalization (RLE) method from *DESeq2* (Anders & Huber, 2010). When both methods were compared with the raw read count distribution per sample, *DESeq2* managed to better standardize the samples in both species (Fig. S2). We then tested for differential expression in both treatment (for all the comparisons between control and an experimental treatment) and colony (for all possible colony pair-wise comparisons) levels using the Wald test method. Genes whose false discovery rate (FDR) was lower than 0.05 were considered as differentially expressed genes (DEGs). We could not test the interactive effects between treatment and colony since the number of replicates per interactive factor level was one. We instead compared which DEGs

overlapped between treatment and colony DEG lists, to consider them as having both treatment and colony effects.

To test whether overlap between two DEG sets is greater than expected by chance, we applied the hypergeometric test using the *phyper* function from R, calculating the following four variables: q (size of overlap, minus one), m (number of DEGs in experiment comparison 1), n (total number of expressed genes, minus m) and k (number of DEGs in comparison experiment 2).

Detecting noise in expression results for *B. occidentalis* data

After observing the clustering of the *B. occidentalis* samples according to their expression with a PCA (Fig. S3), samples from a single colony across all treatments appeared to as outliers that could mask the detection of expression differences of more genes. To explore this issue, we performed the differential expression analysis four additional times, but removing one complete set of samples from a single colony each time. We produced four additional datasets and compared the number of differentially expressed genes obtained per analysis with the data set with all the samples (Table S3). Eliminating colonies 19.244, 19.257 and 19.263 from the dataset did not significantly change the number of differentially expressed genes compared to the results using all the samples, while eliminating the samples from the apparent outlier, colony 19.235, did. Hence, we decided to remove samples from colony 19.235 from our analysis, obtaining a total of 536 DEGs for subsequent analyses.

Testing sample size effect for *B. impatiens* dataset

The differences in total number of differentially expressed genes in *B. impatiens* was an order of magnitude higher than in *B. occidentalis*. We tested whether this difference in

number of DEGs was due to differences in sample size by comparing the results of the differential gene expression analysis using the same number of *B. impatiens* samples than we used for the *B. occidentalis* analysis. We selected randomly three *B. impatiens* colonies and performed differential gene expression analysis with their respective samples, repeating the process three times. We then calculated the mean number of differentially expressed genes per treatment and dataset and compared it with the numbers from the analysis that included all the samples with a hypergeometric test. The mean number of differentially expressed genes per treatment in the reduced data set was significantly different from the number of DEGs found in the analysis with all the *B. impatiens* samples (hypergeometric test P-value < 0.001), thus sample size differences explain some differences in DEG numbers between analyses (Table S2). Nevertheless, when comparing the number of DEGs from the *B. impatiens* iterative dataset with the number of DEGs from *B. occidentalis*, we found that this overlapping gene set was not significantly greater than by chance (hypergeometric test P-value = 1), which suggests that both species had no differences in amplitude of response to the treatments (Table S2).

RNA-seq weighted gene co-expression network analysis pipeline: extended specifications

After filtering and normalization of the reads with *DESeq2* as described above, we analyzed the data with Weighted Gene Co-expression Network Analysis (*WGCNA*) R package (Langfelder & Horvath, 2008). We log-transformed the normalized read counts, adding one to all the counts to avoid problems with the transformation of gene counts with a value of zero, and removed gene entries with variance equals to zero. After exploring the data, we used the soft threshold of seven, proposed by the manual for a sample size between 30 and 40 samples in unsigned networks. We built the co-expression

network and extracted the co-expressed gene modules using the function *blockwiseModules()*, with the parameters *maxBlockSize* = 11000, *networkType* = "unsigned", *power* = 7, *detectCutHeight* = 0.9, *deepSplit* = 4 and *minModuleSize* = 50. We detected the most connected genes within a module for all modules calculating Kleinberg's hub centrality scores using the *hub_score()* function in R and picking the gene with the highest hub score in each module. We used module eigengene values to test the effect of species, treatment, and their interaction, with a PCA (Fig. S7) and with Kruskal-Wallis tests, followed by Dunn's tests for multi-level comparisons. We used the gene list from each module to perform GO term enrichment analyses with *topGO* (Alexa *et al.*, 2006).

Quantitative Polymerase Chain Reaction (qPCR) further specifications

We designed primers with the online version of PrimerBLAST using available genomes from *B. terrestris* (Sadd *et al.*, 2015, for *B. occidentalis*), *B. impatiens* (Sadd *et al.*, 2015), *B. terricola* (Kent *et al.*, 2018) and *B. cullumanus* (Sun *et al.*, 2020, for *B. griseocollis*). We used alignments between *B. terrestris* genome and *B. occidentalis* RNAseq reads to occasionally refine primer design in *B. occidentalis*. If a pair of primers did not work, we tested primers from another species, in case they worked, before designing new ones. We used NCBI Primer-BLAST (Ye *et al.*, 2012) to design the primers on the specific templates. Our NCBI Primer-BLAST custom parameters were 150 bps to 250 bps as product size, 58°C to 62°C as melting temperature (Tm) range, with a Tm value difference not higher than 2°C between forward and reverse primers, and using either *B. impatiens* or *B. terrestris* genomes hosted in NCBI to identify potential unspecific amplification products. We checked each primer pair for hairpin or primer dimer formation with the Thermo Fisher Scientific online tool 'Multi Primer Analyzer'. We designed primers of

four housekeeping genes (*Actin-5c*, *Elongation factor alpha1 copy 2*, *phospholipase A2* and *ribosomal protein large subunit 13*) for all four species. These genes were confirmed as reference genes in qPCRs for bumble bee cDNA in (Horňáková *et al.*, 2010). We then selected a set of 20 differentially expressed genes to be tested in all four species, and designed primers following the instructions above. The list of primers used for each sample and gene is in Table S4.

We used 1 µg total RNA per sample and synthesized cDNA with iScript cDNA kit (BioRad) as DNA template. We tested all the primers on their respective target cDNAs via PCR (95°C for 2 minutes, 35 cycles of 95°C for 30 seconds, 60°C for 30 seconds and 72°C for 30 seconds; 72°C for 5 minutes) and agarose gel (1% w/v) to check the presence of a single amplification product of the right size. We ran the qPCR reactions with 25ng of cDNA in 1µL of nuclease-free water per reaction in *B. impatiens*, *B. griseocollis*, *B. occidentalis* and *B. terricola* using primers for the four housekeeping genes using the Luna qPCR kit (New England Bio Labs), adjusting the reaction volume to 10µL (5µL of Luna qPCR reaction mix, 3.5µL of nuclease-free water, 1µL containing 25ng of cDNA and 0.5 µL of both forward and reverse 10 µM concentrated primers) in 96 well plates (0.1µL of well capacity). Each reaction had two technical replicates per plate. The qPCR conditions were a hot start at 95°C for 1 minute, then 40 cycles at 95°C for 15 seconds and 60°C for 30 seconds each. To assess the integrity of the PCR product, we set a melting curve ranging from 60°C to 95°C with fluorescence reads every increase of 0.3°C. We analyzed the results of the four housekeeping genes with GeNorm v3 (Vandesompele *et al.*, 2002) to determine the most stable expressed gene between samples to be used as a reference gene in each species. We then performed qPCRs for the 20 selected genes of interest as described above and calculated relative quantities (RQs) following the Pfaffl

method (Pfaffl, 2001). We chose this method due to the high differences of primer efficiency values between reference and target genes.

To compare for potential mismatches between RNA-seq and qPCR expression data, we calculated Pearson's correlation coefficient (*r*) between logFC(CPM) from the RNA-seq dataset and logFC(RQ) values from qPCR in each species. These values were $r = 0.88$ for *B. occidentalis* and $r = 0.84$ for *B. impatiens* Fig. S8. When comparing whether the direction of gene expression (upregulated or downregulated) for a specific gene and treatment matched between RNA-seq and qPCR data, we found that 50 out of 60 (83.33%) in both *B. occidentalis* and *B. impatiens* showed a directional match. Among the contrasts that showed opposite expression directions, seven contrasts in *B. occidentalis* and another seven in *B. impatiens* had fold change absolute values between 0 and 1, which indicates that the expression differences were not as strong for these contrasts and the methods could have differences of sensitivity to quantify the gene expression for these cases. We did not account for statistical significance in this analysis because the number of comparisons was different (close to ten thousand in the RNA-seq analysis opposed to 240 comparisons in the qPCR analysis), and thus, the obtained FDR values would not be directly comparable.

Supporting Information References

Alexa, A., Rahnenführer, J., & Lengauer, T. (2006). Improved scoring of functional groups from gene expression data by decorrelating GO graph structure. *Bioinformatics, 22*(13), 1600-1607.

Altschul, S. F., Gish, W., Miller, W., Myers, E. W., & Lipman, D. J. (1990). Basic local alignment search tool. *Journal of molecular biology, 215*(3), 403-410.

Anders, S., & Huber, W. (2010). Differential expression analysis for sequence count data. *Genome biology, 11*(10), R106. doi:10.1186/gb-2010-11-10-r106

Anders, S., Pyl, P. T., & Huber, W. (2014). HTSeq—a Python framework to work with high-throughput sequencing data. *Bioinformatics, 31*(2), 166-169. doi:10.1093/bioinformatics/btu638

Andrews, S. (2010). FastQC: A quality control tool for high throughput sequence data. *Reference Source*.

Bolger, A. M., Lohse, M., & Usadel, B. (2014). Trimmomatic: a flexible trimmer for Illumina sequence data. *Bioinformatics, 30*(15), 2114-2120.

Camacho, C., Coulouris, G., Avagyan, V., Ma, N., Papadopoulos, J., Bealer, K., & Madden, T. L. (2009). BLAST+: architecture and applications. *BMC Bioinformatics, 10*(1), 421. doi:10.1186/1471-2105-10-421

Dobin, A., Davis, C. A., Schlesinger, F., Drenkow, J., Zaleski, C., Jha, S., Batut, P., Chaisson, M., Gingeras, T. R. (2012). STAR: ultrafast universal RNA-seq aligner. *Bioinformatics, 29*(1), 15-21. doi:10.1093/bioinformatics/bts635

Horňáková, D., Matoušková, P., Kindl, J., Valterová, I., & Pichová, I. (2010). Selection of reference genes for real-time polymerase chain reaction analysis in tissues from *Bombus terrestris* and *Bombus lucorum* of different ages. *Analytical Biochemistry, 397*(1), 118-120.

Kent, C. F., Dey, A., Patel, H., Tsvetkov, N., Tiwari, T., MacPhail, V. J., Gobeil, Y., Harpur, B. A., Gurtowski, J., Schatz, M. C. (2018). Conservation genomics of the declining North American bumblebee *Bombus terricola* reveals inbreeding and selection on immune genes. *Frontiers in genetics, 9*, 316.

Langfelder, P., & Horvath, S. (2008). WGCNA: an R package for weighted correlation network analysis. *BMC Bioinformatics, 9*(1), 559. doi:10.1186/1471-2105-9-559

Love, M. I., Huber, W., & Anders, S. (2014). Moderated estimation of fold change and dispersion for RNA-seq data with DESeq2. *Genome biology, 15*(12), 1-21.

Pfaffl, M. W. (2001). A new mathematical model for relative quantification in real-time RT–PCR. *Nucleic acids research, 29*(9), e45-e45.

Robinson, M. D., McCarthy, D. J., & Smyth, G. K. (2009). edgeR: a Bioconductor package for differential expression analysis of digital gene expression data. *Bioinformatics, 26*(1), 139-140. doi:10.1093/bioinformatics/btp616

Sadd, B. M., Barribeau, S. M., Bloch, G., De Graaf, D. C., Dearden, P., Elsik, C. G., Gadau, J., Grimmelikhuijzen, C. J., Hasselmann, M., Lozier, J. D. (2015). The genomes of two key bumblebee species with primitive eusocial organization. *Genome biology, 16*(1), 76.

Sun, C., Huang, J., Wang, Y., Zhao, X., Su, L., Thomas, G. W. C., Zhao, M., Zhang, X., Jungreis, I., Kellis, M., *et al.* (2020). Genus-Wide Characterization of Bumblebee Genomes Provides Insights into Their Evolution and Variation in Ecological and Behavioral Traits. *Molecular Biology and Evolution, 38*(2), 486-501. doi:10.1093/molbev/msaa240

Vandesompele, J., De Preter, K., Pattyn, F., Poppe, B., Van Roy, N., De Paepe, A., & Speleman, F. (2002). Accurate normalization of real-time quantitative RT-PCR

data by geometric averaging of multiple internal control genes. *Genome biology, 3*(7), research0034.0031. doi:10.1186/gb-2002-3-7-research0034

Ye, J., Coulouris, G., Zaretskaya, I., Cutcutache, I., Rozen, S., & Madden, T. L. (2012). Primer-BLAST: A tool to design target-specific primers for polymerase chain reaction. *BMC Bioinformatics, 13*(1), 134. doi:10.1186/1471-2105-13-134

Supporting Information Table Index

Tables in Martin-Blazquez_et_al_SupportingTable[number].xlsx

Table S1. RNA-seq library read number and mapping efficiency statistics.

Table S2. Iterations of differential gene expression analysis in *B. impatiens*.

Table S3. Noise detection in *B. occidentalis* differential gene expression analysis.

Table S4. qPCR primer details.

Table S5. Differentially expressed genes in *B. occidentalis*.

Table S6. Differentially expressed genes in *B. impatiens.*

Table S7. Enriched GO terms in *B. occidentalis*.

Table S8. Enriched GO terms in *B. impatiens*.

Table S9. Gene expression differences between *B. occidentalis* colonies.

Table S10. Gene expression differences between *B. impatiens* colonies.

Table S11. List of genes per gene co-expression network module.

Table S12. Top hub genes per gene co-expression network module.

Table S13. Enriched GO terms per gene co-expression network module.

Table S14. Effect of treatment and species in each gene co-expression network module.

Supporting Information Figures

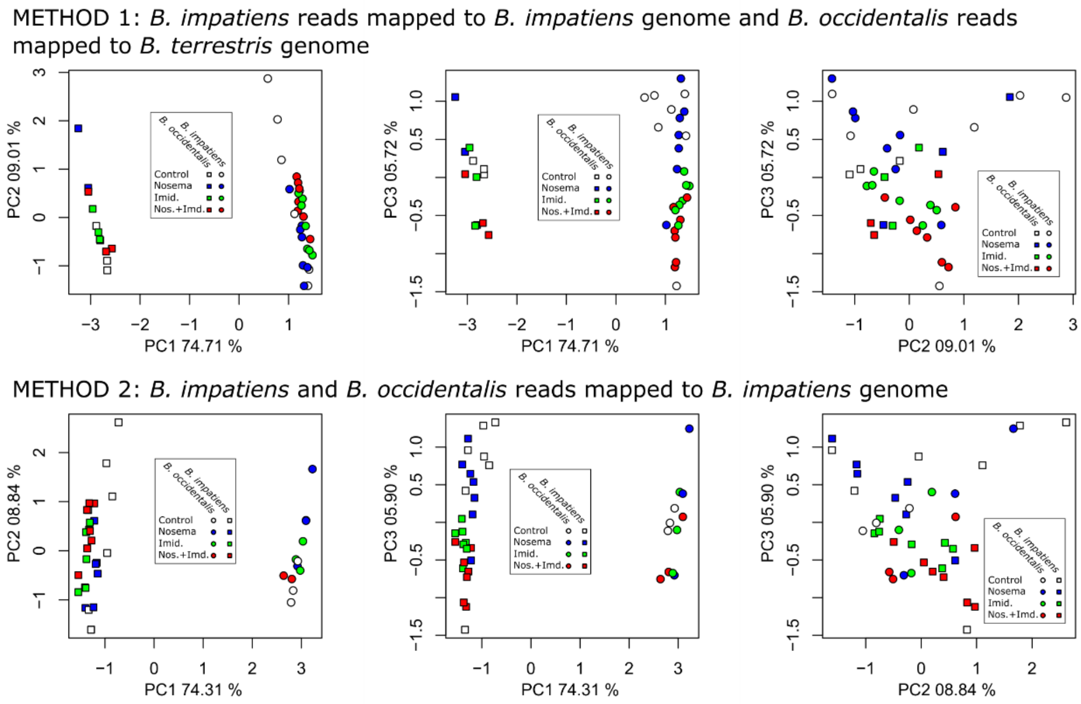

Fig. S1. Comparison between mapping both *B. impatiens* and *B. occidentalis* to a single reference genome or to two separate genomes. Each biplot was generated from a principal component analysis (PCA). The top row shows biplots generated after aligning the *B. occidentalis* reads to *B. terrestris* genome and the *B. impatiens* reads to the *B. impatiens* genome; the bottom row shows biplots generated after aligning both the *B. occidentalis* and *B. impatiens* reads to *B. impatiens* genome. Biplots in the first column show the PC1 and PC2, in the second column the PC1 and PC3, and the third column the PC2 and PC3. Samples in the biplot are shape coded for species (squares: *B. occidentalis*, circles: *B. impatiens*) and color coded for exposure treatment (white: control, blue: *Nosema*, green: imidacloprid, red: imidacloprid and *Nosema*). Each principal component axis label is followed by its percentage of explained variance.

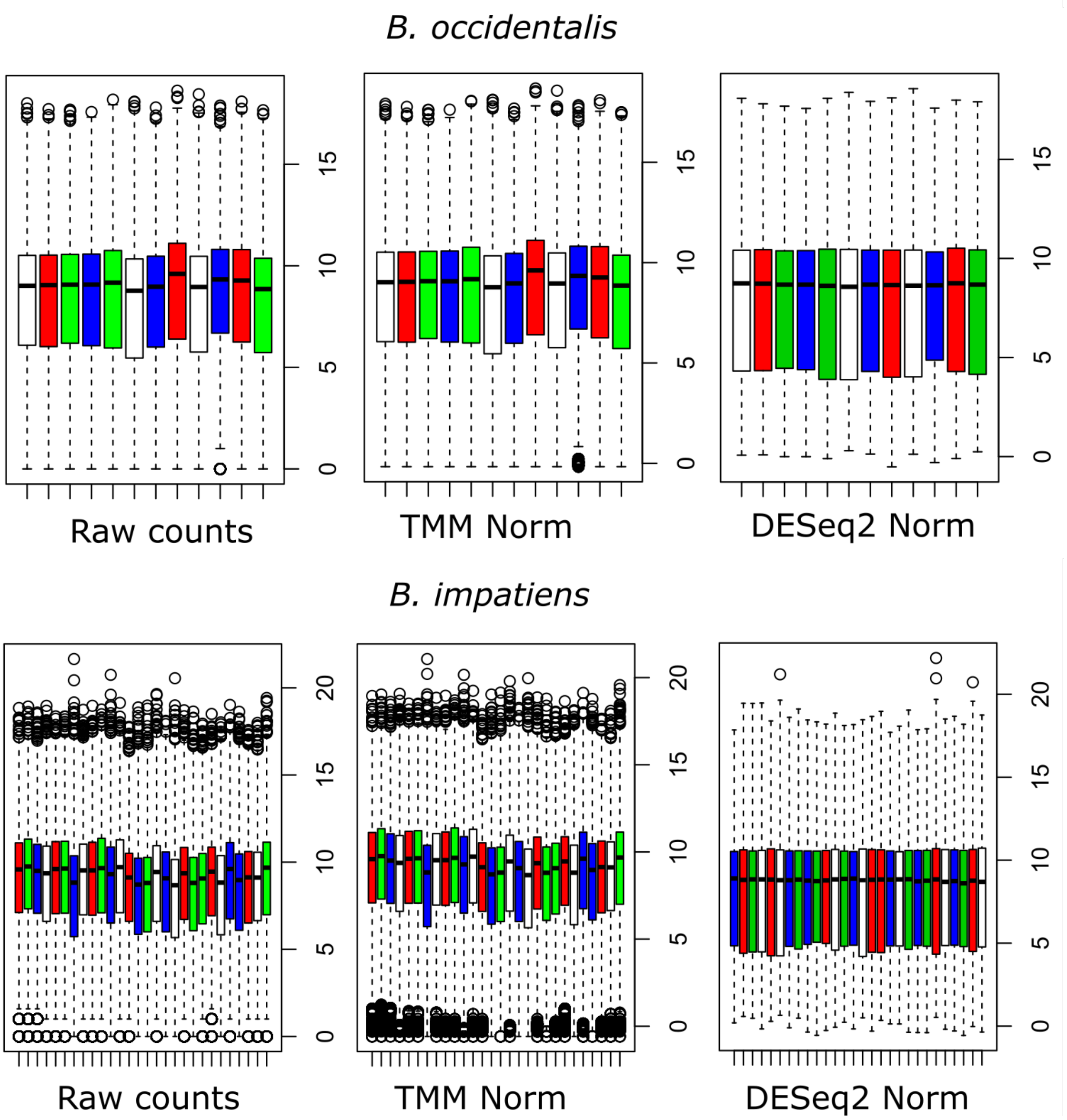

Fig. S2. Comparison of read count normalization methods for *B. occidentalis* and *B. impatiens*. Each boxplot shows the distribution of read counts per gene (log-transformed), with the samples on the x-axis and the read count values on the y-axis. First, second and third columns of plots are raw counts, *edgeR* trimmed-median mean normalized counts, and *DESeq2* normalized counts, respectively. Each box represents a sample color coded according to its exposure treatment (white: control, blue: *Nosema*, green: imidacloprid, red: imidacloprid and *Nosema*).

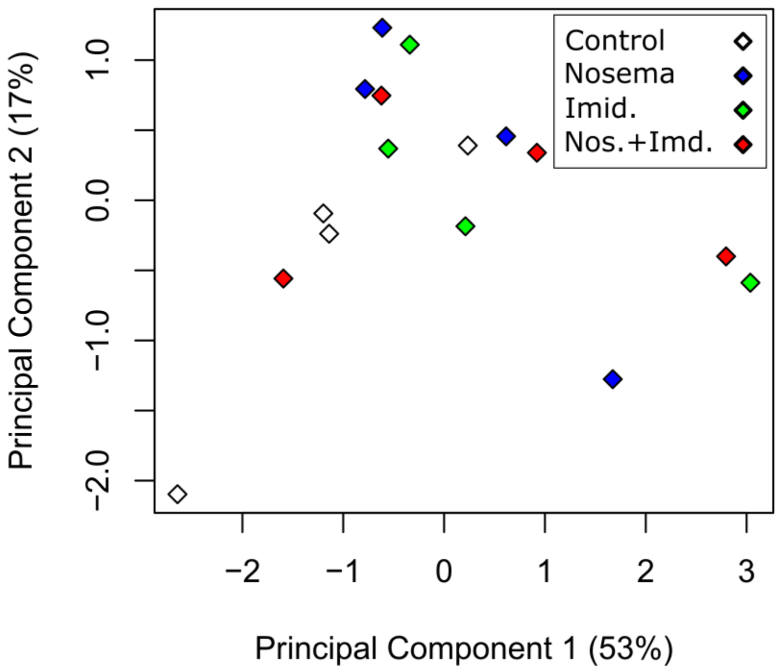

Fig. S3. *B. occidentalis* sample clustering across all colonies. The biplot, generated from principal component analysis (PCA) of the gene expression counts from the 500 most expressed genes in all the *B. occidentalis* samples, shows the PC1 and the PC2. Following each PC axis label in parentheses is the percentage of variance explained by the PC. Each diamond represents a sample, color coded according to its exposure treatment (white: control, blue: *Nosema*, green: imidacloprid, red: imidacloprid and *Nosema*).

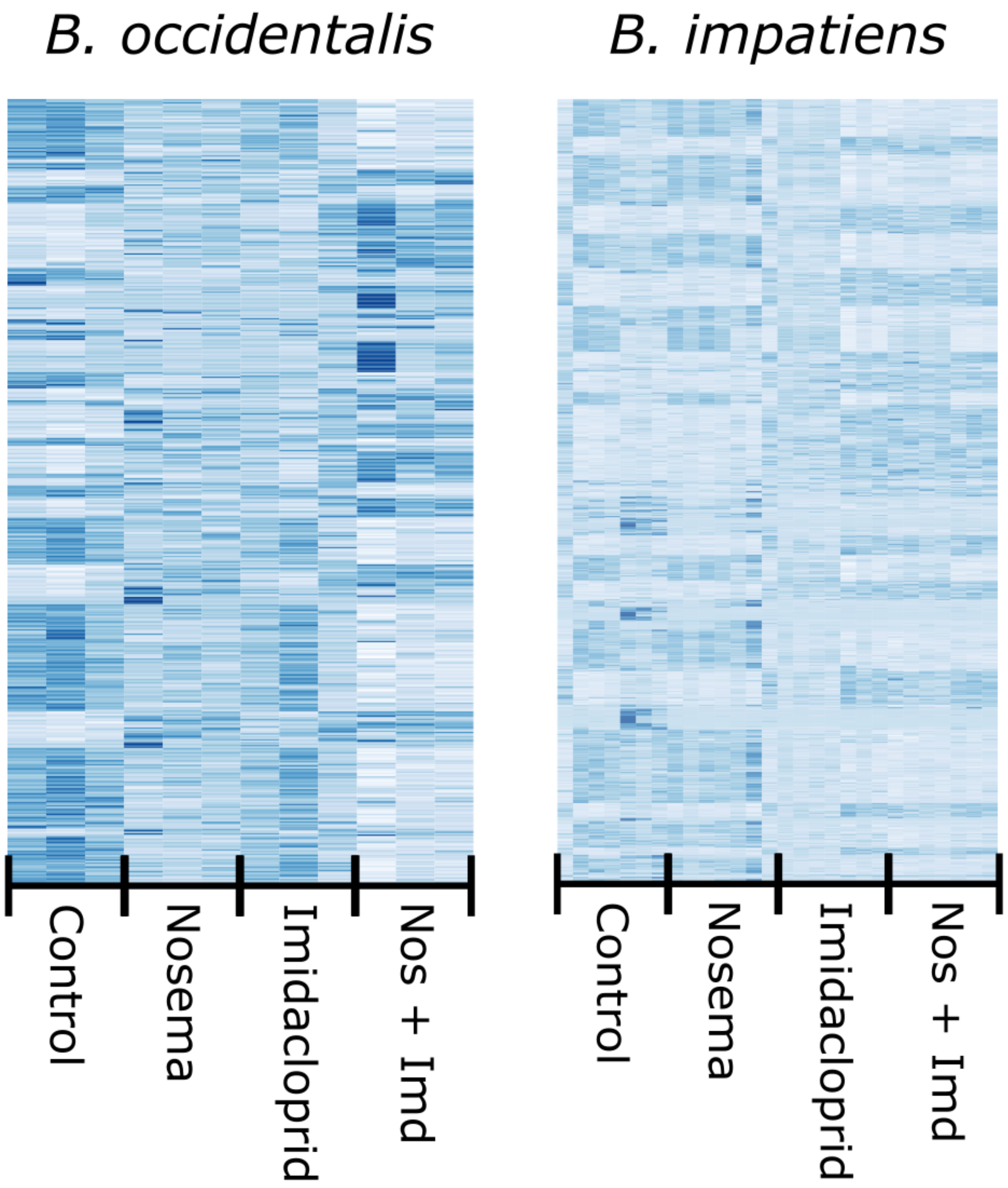

Fig. S4. Differentially expressed genes expression profile for *B. occidentalis* (left) and *B. impatiens* (right) exposed to different treatments of imidacloprid and *Nosema*. Each line on the y-axis represents the DEGs with the intensity of the tile is proportional to the normalized read counts (counts per million mapped reads (CPMs), but standardized to a mean of zero and a standard deviation of 1, with light blue representing low and dark blue representing high normalized read counts). Gene clustering was produced by scaled CPM values.

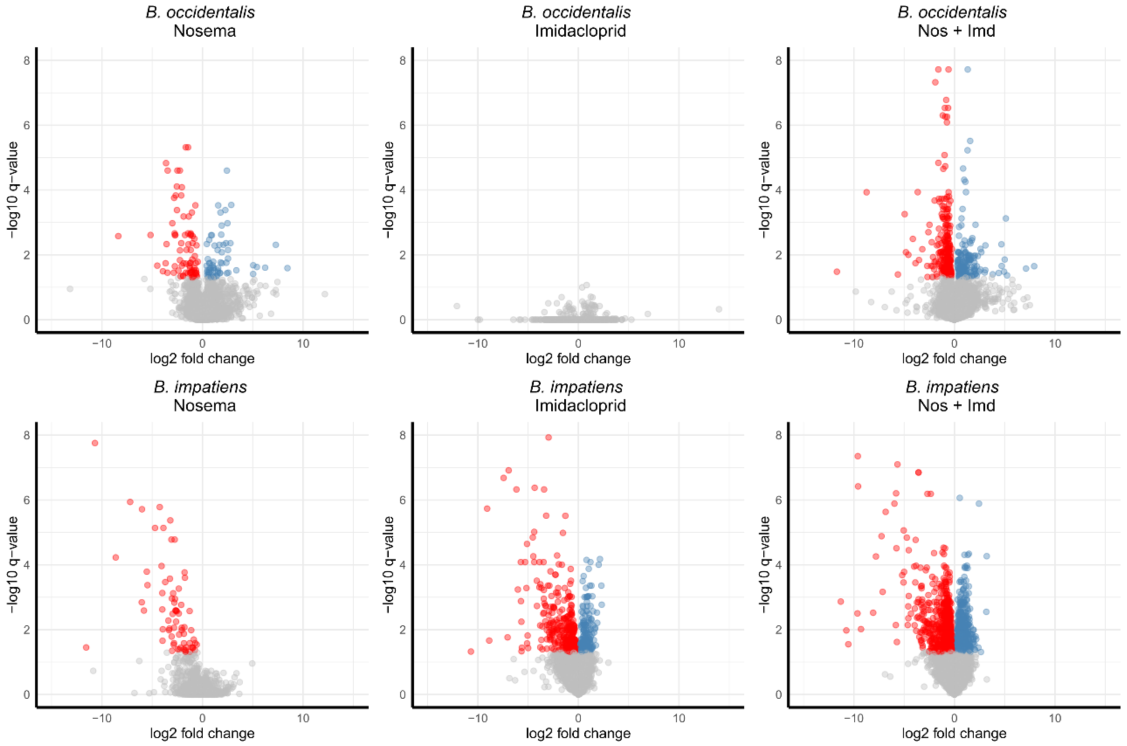

Fig. S5. Volcano plots of differential gene expression analysis for *B. occidentalis* (top) and *B. impatiens* (bottom) exposed to different treatments of imidacloprid and *Nosema* relative to control. The x-axis shows the logarithm base 2 of the fold change (logFC) and the y-axis the negative logarithm base 10 of the false discovery ratio (logFDR). Red dots are downregulated genes, blue dots are upregulated genes and grey dots are genes without significant differential expression.

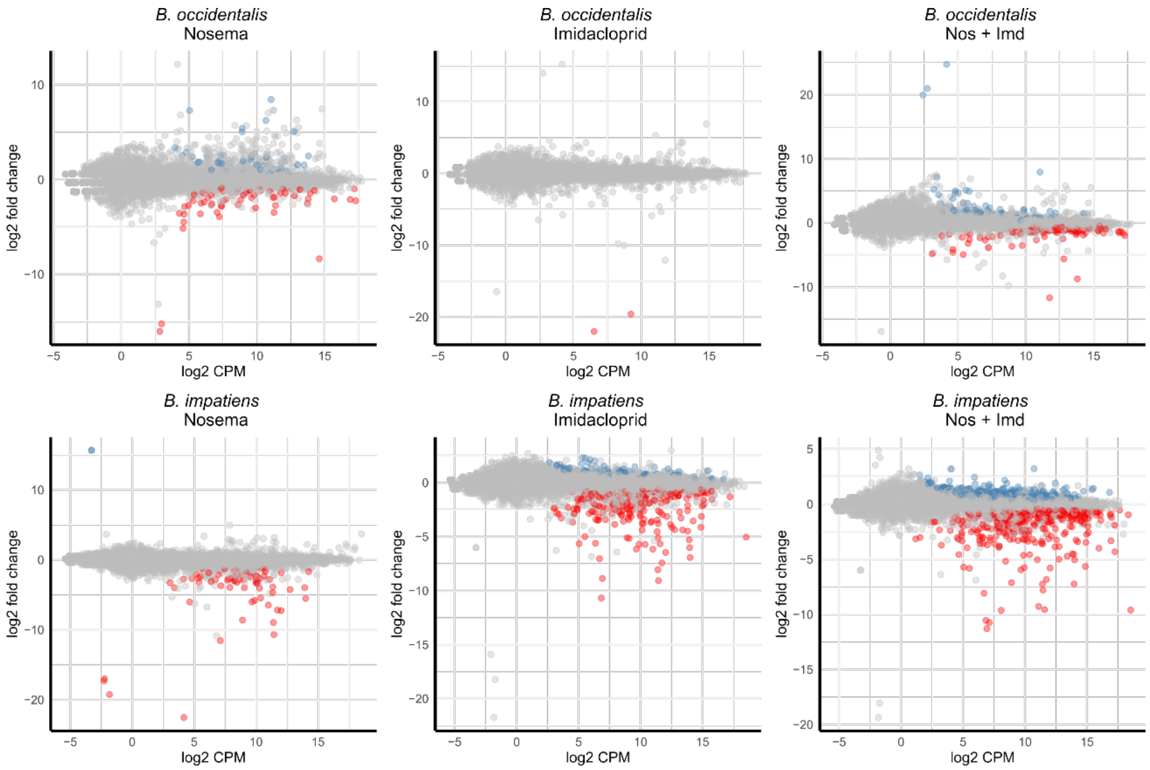

Fig. S6. MA plots of differential gene expression analysis for *B. occidentalis* (top) and *B. impatiens* (bottom) exposed to different treatments of imidacloprid and *Nosema* relative to control. The x-axis shows the logarithm to the base 2 of the mean normalized counts for a gene (log2 CPM) and the y-axis shows the logarithm base 2 of the fold change (log2 fold change). Red dots are downregulated genes, blue dots are upregulated genes and grey dots are genes without differential expression.

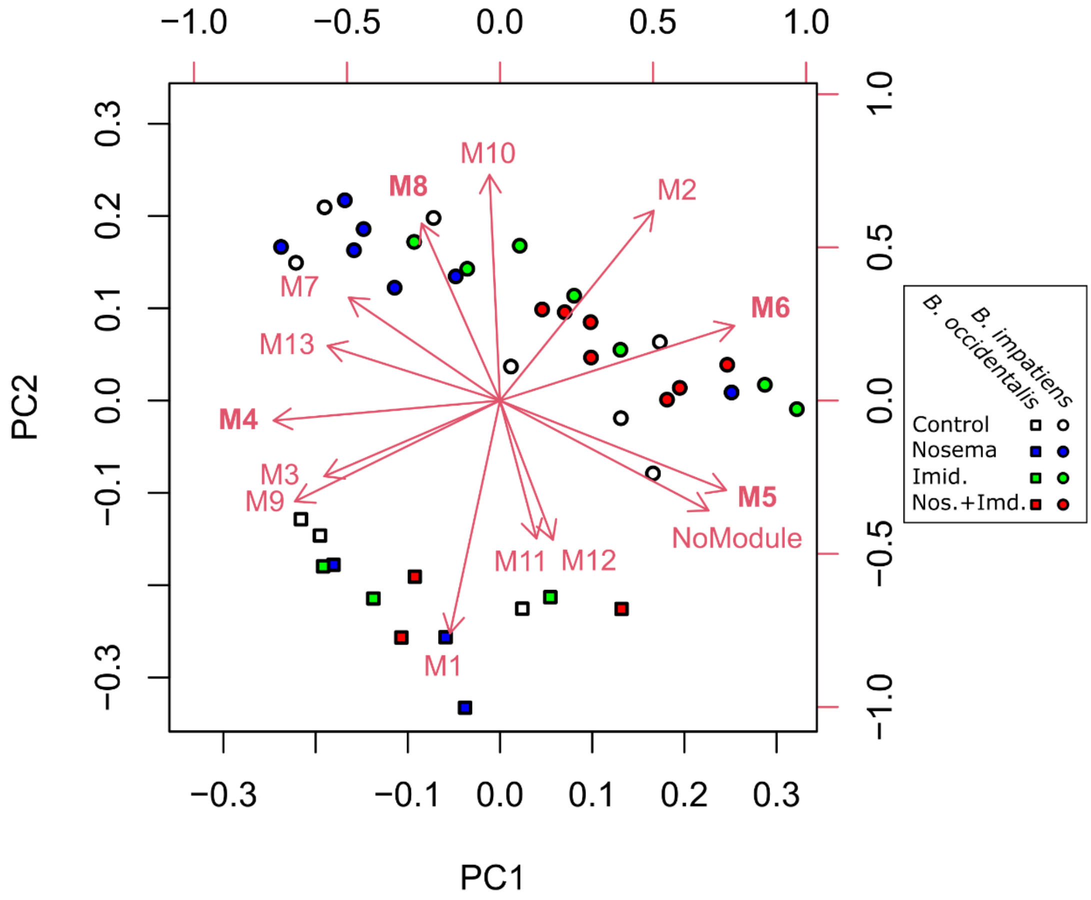

Fig. S7. Co-expressed gene modules and sample clustering across species (*B. occidentalis* and *B. impatiens*) exposed to different treatments of imidacloprid and *Nosema*. The biplot shows the PC1 and the PC2 from principal component analysis (PCA) of the module eigengenes (ME) from all the modules generated by the weighted gene co-expression network analysis (WGCNA). Samples are represented as squares (*B. occidentalis*) and circles (*B. impatiens*), and are color coded according to treatment (white: control, blue: *Nosema*, green: imidacloprid, red: imidacloprid and *Nosema*). Red arrows pointing to a module name shows the position of each ME in the biplot, modules significant for treatment effects in bold.

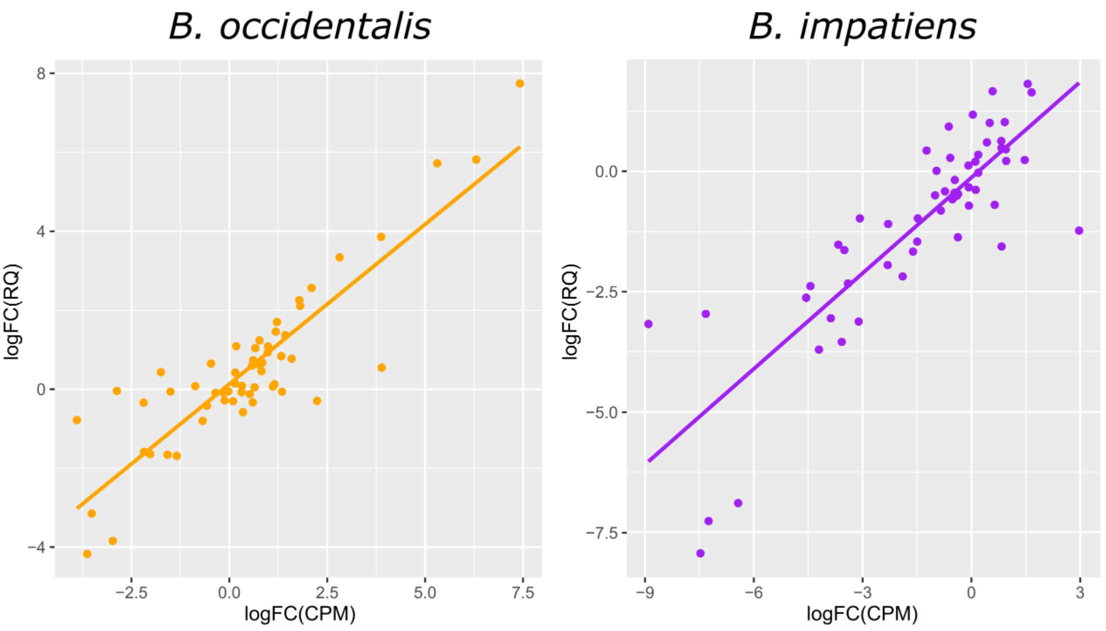

Fig. S8. Correlations between RNA-seq and qPCR data. The x-axis shows the logarithm to the base two of the fold change of the normalized counts as read counts per million mapped reads (CPM) from the RNA-seq analysis. The y-axis shows the logarithm to the base two of the fold change of the relative quantity (RQ) from the qPCR analysis. The first plot shows the data from *B. occidentalis* (Pearson's correlation coefficient $r = 0.88$, P-value < 0.001), the second shows the data from *B. impatiens* (Pearson's correlation coefficient $r = 0.84$, P-value < 0.001).
